# Case report: Accelerated regression of giant cardiac rhabdomyomas in neonates with low dose everolimus

**DOI:** 10.3389/fped.2023.1109646

**Published:** 2023-02-15

**Authors:** Daniel Hurtado-Sierra, Judy Ximena Ramos Garzón, Lyda Z. Rojas, Oscar Fernández-Gómez, Francisco Manrique-Rincón

**Affiliations:** ^1^ Pediatric Cardiology Unit, Instituto del Corazón de Bucaramanga, Bucaramanga, Colombia; ^2^ Nursing School, Universidad Industrial de Santander, Bucaramanga, Colombia; ^3^ Research Center, Fundación Cardiovascular de Colombia, Floridablanca, Colombia

**Keywords:** cardiac tumors, rhabdomyoma, tuberous sclerosis, everolimus, infant, newborn

## Abstract

Cardiac rhabdomyoma (CRHM) is the principal cardiac tumor in children and is most often associated with tuberous sclerosis complex (TSC). Mutations in the *TSC1* and *TSC2* genes cause the overactivation of the mammalian Target of Rapamycin (mTOR). This protein family is responsible for abnormal cell proliferation leading to the formation of CRHMs and hamartomas in other organs. Despite the tendency for spontaneous regression, some CRHMs can cause heart failure and intractable arrhythmias, requiring surgical resection. In recent years, the use of everolimus and sirolimus (mTOR inhibitors) in the treatment of CRHMs has been reported. We report two cases of neonates with giant rhabdomyomas, with hemodynamic repercussions treated with low-dose everolimus (4.5 mg/m^2^/week). In both cases, we obtained an approximate decrease of 50% in the total area of the mass after three weeks of treatment. Despite rebound growth after stopping the drug, we were able to evidence that the use of low doses of everolimus immediately after birth is effective and safe in the treatment of giant CRHMs, avoiding surgical resection of the tumor and associated morbidity and mortality.

## Introduction

Cardiac Rhabdomyoma (CRHM) is the main primary cardiac tumor diagnosed in children. It represents more than 60% of all cardiac tumors in this population group, with an approximate cumulative incidence ranging between 0.02 and 0.08% in newborns (1, 2). CRHMs are well-defined masses of enlarged myocytes with scattered myofilaments, vacuolized cytoplasm, and abundant glycogen stores (3). Immunohistochemical studies have shown that CRHMs do not express cell proliferation markers, due to the above, they are not considered true neoplasms. On the contrary, many authors describe them as hamartomas composed of striated muscle fibers that appear only in the heart (2–4).

CRHMs are associated with tuberous sclerosis complex (TSC) in 60 to 80% of cases (2), an autosomal dominant disease that causes the growth of hamartomas in multiple organs such as the skin, central nervous system, kidneys, lungs, liver, and heart (5). 85% of patients with TSC have mutations that inactivate the genes that encode the proteins hamartin (TSC1) or tuberin (TSC2). These inhibit the mammalian Target of Rapamycin (mTOR), a family of serine-threonine-kinase proteins that participates in the regulation of cell growth, proliferation, differentiation, and metabolism (6). Thus, mTOR overactivation is responsible for abnormal cell proliferation leading to the formation of hamartomas in the heart and other organs. CRHMs are located mainly in the ventricles at the intracavitary or intramural level and less frequently in the atria (1, 2). Most CRHMs present as multiple masses, a characteristic that increases the possibility of a diagnosis of TSC. This can be corroborated during the fetal period, where multiple CRHMs are the earliest manifestation of TSC (1, 7).

Fetal CRHMs can usually be detected with ultrasound from the 20th week of gestation, at which time they begin to increase in size in response to transplacental maternal estrogens (8). After the 32nd week of gestation, the growth of the CRHM slows down, and at birth, the mass regression process begins as the mitotic potential of maternal estrogens is lost (2). Complete regression of CRHMs occurs in most cases during early childhood, being obtained in up to 50% of patients in the first two years of life (1, 7–11). Most of the patients with cRHMs remain asymptomatic and do not require treatment. However, despite the good results and the capacity for spontaneous regression, these masses can cause heart failure due to obstruction of the inlet or outlet tracts of the heart ventricles, interference with ventricular contraction due to large intramural masses, and difficult-to-control arrhythmias (1, 7, 10). Clinically significant arrhythmias affect up to 16% of patients. There are conduction disorders due to dysfunction of the sinus node and atrioventricular (AV) node; to tachyarrhythmias such as ectopic atrial tachycardia, reentry in accessory AV pathway (Wolff-Parkinson-White syndrome), and ventricular tachycardia (12–15).

Hemodynamic complications associated with cRHMs are the principal cause of death in patients with TSC before ten years of age (7, 10). Death in the fetal and early neonatal period can result from incessant arrhythmias or the obstructive effect of large masses leading to cardiogenic shock or fetal hydrops (15–17). A rare cause of death is acute myocardial infarction secondary to extrinsic obstruction of the coronary arteries (2, 18).

Surgical treatment is reserved for large cRHMs with severe obstructive effects or arrhythmias that do not respond to drug treatment (7). However, surgical resection is associated with potential complications and a significant risk of death (7, 19–23). In some cases, the masses are inoperable due to their large size, location, multiplicity, or due to conditions specific to the patient, such as critical condition, low weight, or prematurity (7, 17, 19, 22, 23). Partial surgical resection of CRHMs is an acceptable measure to reduce postoperative complications and associated mortality (2, 23, 24).

Progress in understanding the role of mTOR in cell overgrowth and proliferation has led to the use of mTOR inhibitors (everolimus and sirolimus) as a therapeutic option (off-label) for the management of CRHMs in recent years (6, 7, 9, 19, 22, 25, 26). mTOR inhibitors are effective in other TSC-related tumors, including subependymal giant cell astrocytoma (SEGA) and renal angiomyolipomas. These are also effective for TSC complications such as epilepsy, lymphangioleiomyomatosis, and skin lesions (27–30). To date, there are no results from randomized clinical trials evaluating the efficacy of everolimus in patients with symptomatic CRHMs (19, 22). We report the effect of low-dose everolimus for the treatment of giant CRHMs in the right ventricle with significant hemodynamic compromise in two newborns.

## Case 1

A Female neonate was delivered by cesarean section at 37.4 weeks to an 18-year-old woman with poor prenatal care and no family history of TSC. The fetal echocardiogram had previously demonstrated a large intracardiac mass. At birth, with a weight of 2,890 grams, a height of 48 cm, and an Apgar score of 8 and 9 at 1 and 5 min, respectively.

**Figure 1:**
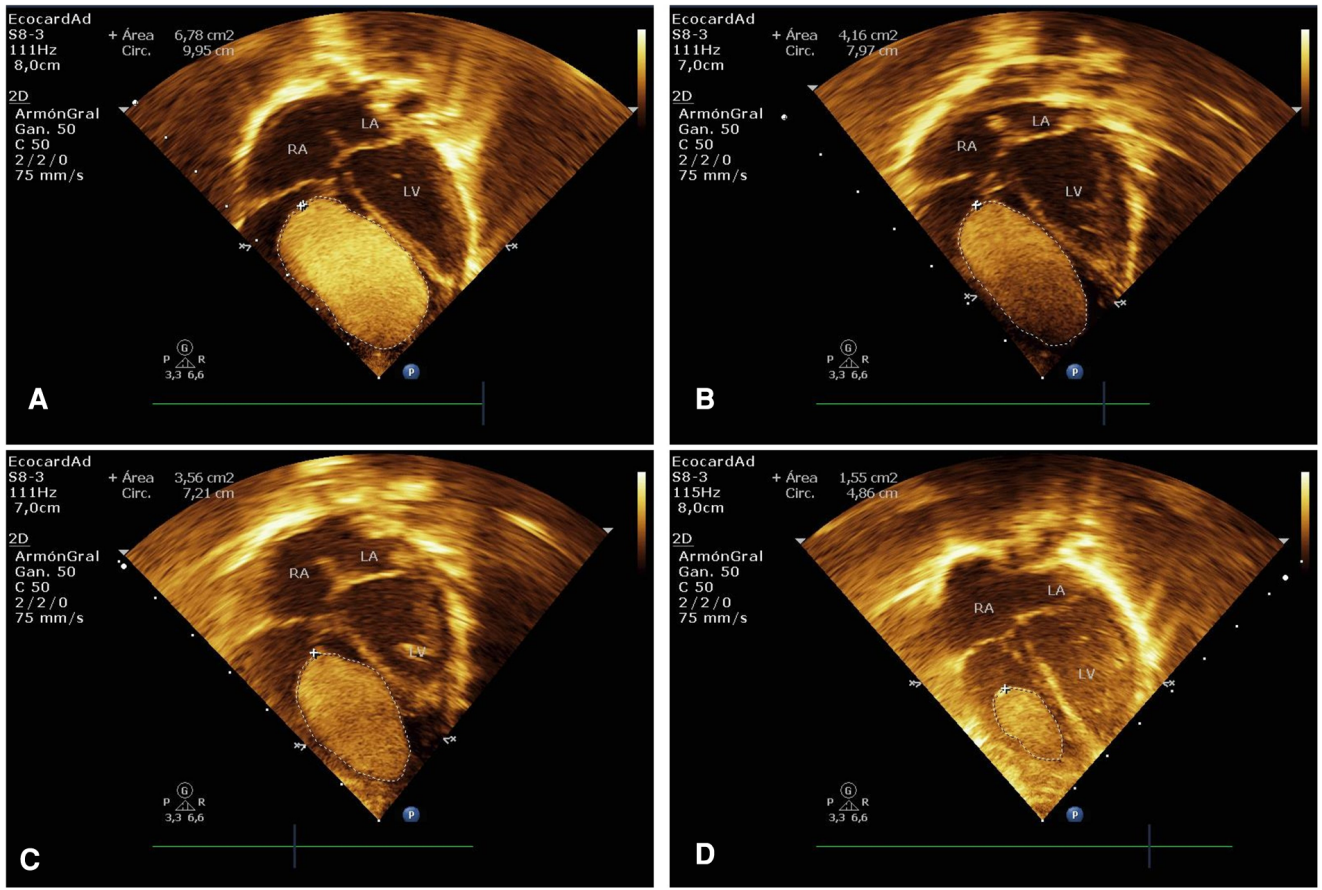
Case 1. (**A**) Immediate postnatal transthoracic echocardiogram in apical 4-chamber view showing a large hyperechogenic and homogeneous mass of 37 mm × 20.7 mm in size (area of 6.78 cm^2^), which occupies a large part of the right ventricular cavity, compatible with a giant rhabdomyoma. (**B–D**) Echocardiographic control at 2, 3, and 13 weeks after treatment with everolimus showed a significant reduction in the size of the rhabdomyoma.

On initial physical examination with rhythmic heart sounds without significant murmurs. She presented mild signs of respiratory distress with a response to low-flow oxygen. The postnatal transthoracic echocardiogram showed a giant mass of 37 mm × 20.7 mm and an area of 6.78 cm^2^ compatible with CRHM. This mass was located in a significant part of the right ventricular cavity, limiting the opening of the tricuspid valve and slightly obstructing the right ventricular outflow tract (Figure 1A). The ductus arteriosus had a shunt from left to right, and the foramen ovale had a bidirectional shunt. The estimated right ventricular systolic pressure was 43 mmHg. The electrocardiogram revealed sinus rhythm with an increased voltage of R waves in right precordial leads and prolonged QRS. No alterations were found in the transfontanellar and renal ultrasound.

After discussing the case with a medical board and because of the significant morbidity and mortality associated with surgical resection of the tumor, the mother signed the informed consent, and oral treatment with everolimus at a low dose of 0.1 mg per day was started on the third day of life. The reported serum level of everolimus at seven days of treatment was 11.5 ng/ml (therapeutic range 5–15 ng/ml) (27, 28, 31). There were no alterations in the initial laboratories (complete blood count, renal function, electrolytes, liver function, glucose, and lipid profile). The newborn remained stable without requiring inotropic or ventilatory support, even after verifying the spontaneous closure of the ductus arteriosus, leaving the hospital at 14 days of life to continue outpatient treatment.

**Figure 2:**
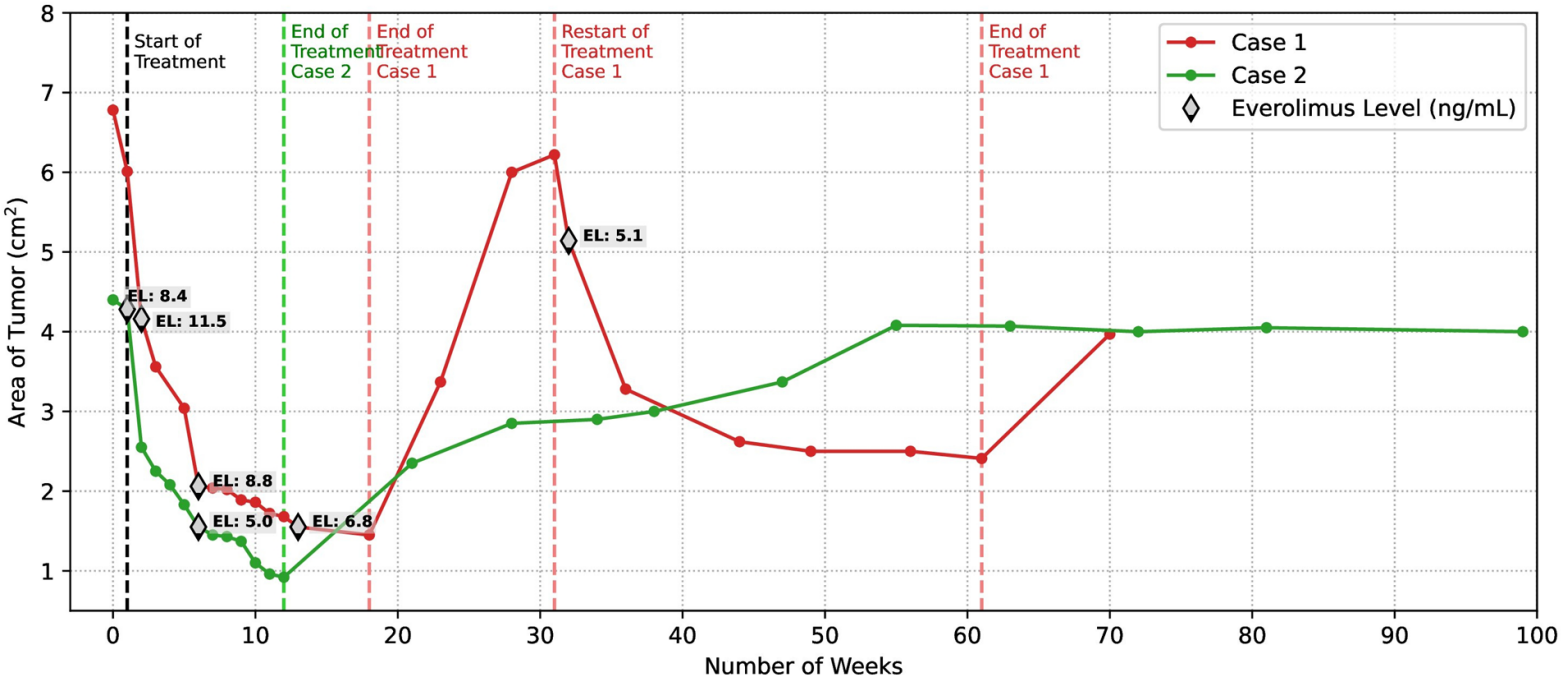
Comparative regression in the size of CRHMs after initiation of treatment with low dose everolimus. An accelerated decrease of close to 50% of the initial area of the CRHM is evident in both cases at three weeks of treatment. The maximum reduction in the mass area was reached at 17 weeks of treatment for case 1 and 12 weeks of treatment for case 2, the two of them close to 79%. In both patients, the serum everolimus level was maintained in therapeutic ranges (target through concentrations between 5 and 15 ng/ml). Once treatment was discontinued CRHM experienced rebound growth, being particularly accelerated in case 1. The restart of everolimus in case 1 demonstrated a rapid reduction in mass size. Case 2 CRHM maintained a stable size without signs of hemodynamic repercussions after undergoing rebound growth.

Echocardiographic follow-up demonstrated a reduction in the mass area of 11.3%, 38.6%, and 47.4% in the first, second, and third weeks, respectively Figures 1B,C, 2). She received everolimus for 17 weeks (121 days), obtaining a reduction in the total area of the mass of 78.6%. The serum level of everolimus remained within the therapeutic range with values of 8.8 ng/ml and 6.7 ng/ml, measured at weeks 5 and 12, respectively. A rebound effect with an increase in the size of the CRHM was observed after discontinuation of everolimus, reaching 49.7% of the initial area 35 days after the end of treatment and 91.7% at 90 days. Simultaneously, a limitation was found in the opening of the tricuspid valve, associated with a progressive increase in right intraventricular pressure. It was decided to start the second cycle of everolimus at twice the dose (0.2 mg per day). A 17.3% decrease in the size of the CRHM was observed in the second week and 47.2% in the sixth week, with everolimus serum levels within the therapeutic range (5.08 ng/mL). A total decrease in the mass area of 61.2% was obtained after 33 weeks (232 days) with the second course of everolimus. Subsequently, the treatment was discontinued (Figure 2).

Adverse effects attributed to everolimus were mild anemia, hypercholesterolemia, and hypertriglyceridemia detected after the fifth week of the first treatment cycle. Anemia was treated with ferrous sulfate, while dyslipidemia was kept under surveillance. The result of the genetic testing for pathogenic variants *TSC1* or *TSC2* was negative, and there were no alterations in the brain magnetic resonance imaging. The echocardiogram performed 42 days after the end of the second treatment cycle showed a new increase in the size of the CRHM, reaching 58.5% of the initial area, without compromise in the opening of the tricuspid valve or clinical manifestations attributed to the CRHM. It was not possible to continue the clinical follow-up of the patient because she was transferred to another hospital.

## Case 2

**Figure 3:**
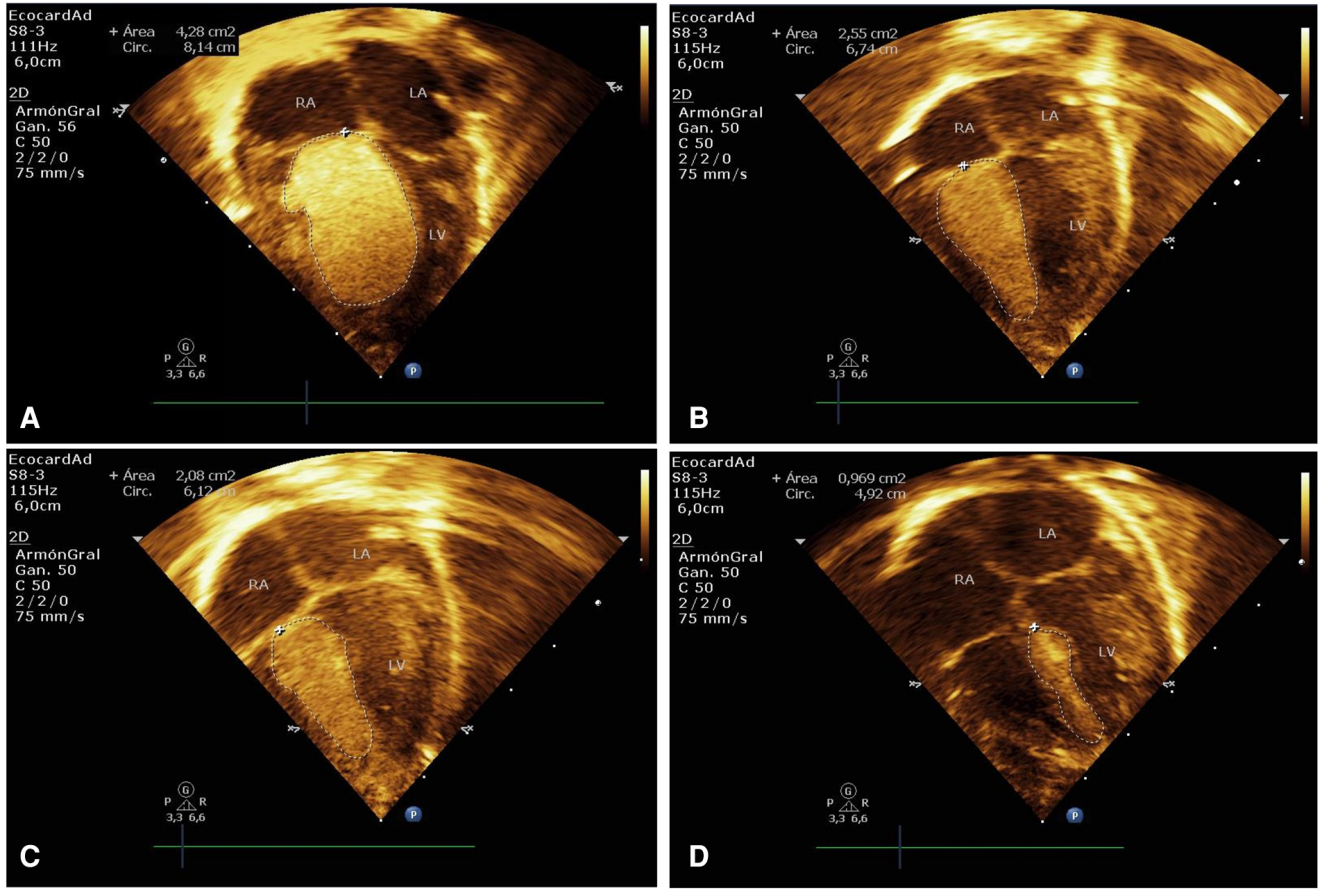
Case 2. (**A**) Immediate postnatal transthoracic echocardiogram in apical 4-chamber projection showing a giant rhabdomyoma measuring 30 mm × 17.3 mm (area of 4.4 cm^2^), adhered to the interventricular septum, and occupying a large part of the right ventricular cavity. (**B–D**) Echocardiographic control at 2, 4, and 11 weeks of treatment with everolimus, showed a significant reduction in the size of the rhabdomyoma.

A male neonate was born by cesarean section at 36.1 weeks to an 18-year-old woman with no family history of TSC. The fetal echocardiogram had previously demonstrated a large intracardiac mass. Birth weight 2,240 grams, length 45 cm, and Apgar scores 7 and 8 at 1 and 5 min, respectively. Initial physical examination evidenced rhythmic heart sounds without significant murmurs. He presented moderate signs of respiratory distress requiring orotracheal intubation and transfer to the neonatal intensive care unit (NICU). The immediate postnatal transthoracic echocardiogram showed a giant mass of 30 mm × 17.3 mm and an area of 4.4 cm^2^ compatible with CRHM. It was adhered to the interventricular septum, occupying a large part of the right ventricular cavity, limiting the opening of the tricuspid valve and displacing the interventricular septum towards the left ventricle, without outflow tract obstruction (Figure 3A). Other rhabdomyomas were found inside the left ventricle, the largest of them measured 17 mm × 8.7 mm, and were adhered to the free wall with an extension to the apex. There was a left-to-right shunt through the ductus arteriosus and foramen ovale. He did not require inotropic support and was extubated 24 h after admission to the NICU, remaining hemodynamically stable even after spontaneous closure of the ductus arteriosus was verified.

Transfontanelar ultrasound performed on the first day of life reported an image like a tuber at the level of the left caudothalamic groove. A brain tomography confirmed the presence of a hyperdense nodule of 7.5 mm in the left caudothalamic groove, without being able to differentiate between a tuber and a SEGA. Renal ultrasound was normal.

After discussing the case with the medical board, considering the significant morbi-mortality associated with surgical resection of the CRHM and with the informed consent of the mother, it was decided to initiate oral treatment with everolimus at a low dose of 0.1 mg per day on the second day of life. The serum everolimus level reported at 6 days of treatment was 8.4 ng/ml. No alterations were found in the hepatic, renal, hematological, and metabolic function tests performed during the first week of treatment. During his hospital stay, he presented several episodes of generalized hypertonia with an abnormal electroencephalogram, and treatment with phenobarbital was initiated. The Holter performed before hospital discharge evidenced ectopic atrial rhythm with no other findings. Once hemodynamic and neurological stability was verified, the patient was discharged at 14 days of life to continue outpatient treatment.

Echocardiographic follow-up showed a reduction in the mass area of 2.8%, 42.1%, and 48.9% in the first, second, and third weeks, respectively (Figures 2, 3B,C). He received everolimus for 12 weeks (84 days), obtaining a reduction in the total mass area of 79.1%. Serum everolimus level remained within the therapeutic range with values of 8.4 ng/ml and 5 ng/ml, measured at weeks 1 and 7, respectively. He presented mild dyslipidemia from the third week and mild anemia from the seventh week of treatment. The Holter control performed at two months of life showed baseline sinus rhythm with prolongation of the QTc interval up to 490 ms, requiring treatment with propranolol for 3 months until normalization of the QTc interval was verified. Genetic testing identified a heterozygous pathogenic variant in the *TSC2* gene.

A rebound was observed with an increase in the size of the CRHM after discontinuation of everolimus, reaching 53.4% of the initial area after 64 days and 64.7% after 112 days. No limitation was found in the opening of the tricuspid valve nor obstruction of the right ventricular outflow tract; therefore, it was not considered necessary to initiate the second cycle of everolimus. Ten months after the end of treatment, the CRHM reached 92.7% of the initial area, with no changes in size or signs of hemodynamic compromise since then (Figure 2).

## Discussion

Due to the low incidence of giant CRHMs, only case reports on the effectiveness and safety of everolimus or sirolimus in the regression of this type of masses are currently available in the literature. The dose, age of initiation, and duration of treatment vary widely among reports, making it difficult to standardize a therapeutic scheme (22). On the other hand, because of the low activity of the CYP3A4 liver enzyme in the neonatal period, this group of patients is at higher risk of exceeding the therapeutic level of the drug and therefore its adverse effects (32). The dose used in our two cases was 4.5 mg/m^2^/week (0.1 mg/day), one-seventh of the dose approved by the FDA (Food and Drug Administration) and EMA (European Medicines Agency) for the treatment of SEGA and drug-resistant focal onset seizures associated with TSC (27, 31, 33). With this dose, we were able to maintain the everolimus serum level within the therapeutic range, with mild adverse effects (34). Likewise, in the systematic review by Sugalska et al., 91.3% of the neonates treated with everolimus received a dose 3 to 7 times lower than the one used for the treatment of SEGAs, however, 14.3% had serum drug levels above the target through concentration (22).

The percentage of decrease in size of CRHMs is not available in most reports of neonates treated with everolimus. In our two cases, we obtained an approximate 50% decrease in the total mass area at 3 weeks of treatment (Figure 2). Declines as rapid as 30% and 50% have been reported in the literature after 11 and 22 days of everolimus treatment, respectively (17, 35).

In the review by Sugalska et al., the average duration of treatment with everolimus or sirolimus was 16 weeks (range 28 to 390 days) (22). Despite receiving treatments of different duration (17 weeks for case 1 and 12 weeks for case 2), the two patients experienced a maximal reduction in the total mass area by close to 79%. The larger size of the mass in case 1 likely required a longer course to achieve the reduction obtained in case 2.

Few articles report long-term follow-up or the possibility of rebound growth of CRHMs after treatment is completed. In the review by Sugalska et al. long-term follow-up barely reached 29.3%, rebound growth of cRHMs was 14.3% in the everolimus group and 13.2% for the sirolimus group (22). We observed a rebound effect in the size of the CRHMs after discontinuation of treatment in both cases, with a particularly accelerated increase in case 1 (Figure 2), which in association with the hemodynamic changes found in the echocardiogram led to the restart of everolimus in this case. Once again, we were able to observe a rapid decrease in the size of the CRHM with the second cycle of treatment, demonstrating the suitability of everolimus in this pathology. On the other hand, the poor long-term follow-up of patients treated with mTOR inhibitors may explain the small number of rebound cases reported in the literature. From all the above, we can conclude that the duration of treatment should be individualized and should be guided not only by the decrease in mass size but also by the improvement of clinical symptomatology and the resolution of hemodynamic alterations generated by CRHM.

Despite the rebound growth observed in the two cases after discontinuation of treatment, we were able to demonstrate that the use of everolimus immediately after birth enables us to overcome the period of greatest hemodynamic lability, avoiding surgical resection of the tumor and the associated morbi-mortality. On the other hand, the natural tendency of CRHMs to involute with age makes rebound growth less relevant.

Without ignoring the efficacy of sirolimus in the treatment of some manifestations of TSC (36–39), we decided to use everolimus encouraged by the greater number of randomized clinical trials that have proven the efficacy of this drug in TSC (27–30). On the other hand, everolimus has greater oral bioavailability and reaches a steady state faster than sirolimus (40). The high cost of everolimus and the problems related to its affordability in our environment were permanent limitations. However, this was managed to be overcome to ensure uninterrupted treatment. It is necessary to mention that a disadvantage of everolimus is the lack of an oral solution for use in infants and young children, avoiding crushing and diluting the tablets, thus reducing the risk of inaccurate dosing.

In our case, the use of low doses of everolimus was effective and safe in the treatment of neonates with giant CRHMs and should be considered a reasonable alternative to surgical resection. Randomized clinical trials are needed to support the efficacy of mTOR inhibitors in the treatment of CRHMs. Recently, Stelmaszewski et al. published the protocol of the randomized, placebo-controlled, double-blind, multicenter phase II clinical trial of everolimus in the treatment of patients with symptomatic CRHMs with TSC (ORACLE) (19). This clinical trial is expected to provide further evidence of the effectiveness of everolimus in the treatment of this pathology.

## Data Availability

The original contributions presented in the study are included in the article/Supplementary Material, further inquiries can be directed to the corresponding author/s.
